# Practice pointer: care of men with cancer-predisposing *BRCA* variants

**DOI:** 10.1136/bmj.n2376

**Published:** 2021-10-14

**Authors:** Rachel Horton, Paul Pharoah, Judith Hayward, Anneke Lucassen

**Affiliations:** Clinical Ethics and Law, Faculty of Medicine, University of Southampton, Wessex Clinical Genetics Service, Princess Anne Hospital, Southampton; Department of Public Health and Primary Care, Department of Oncology, Cambridge Cancer Centre, University of Cambridge; Yorkshire Regional Genetics Service, Primary Care Adviser to Health Education England Genomics Education Programme, RCGP Joint Clinical Champion in Genomics Medicine with Dr. Imran Rafi, GP, Shipley Medical Practice, Affinity Care; Clinical Ethics and Law, Faculty of Medicine, University of Southampton and Wellcome Centre for Human Genetics/Director of Centre for Personalised Medicine, University of Oxford

**Keywords:** genetics, genomics, BRCA, screening

Around 1 in 260 men (~0.4%) inherits a cancer-predisposing *BRCA* variant that increases their risk of developing prostate, pancreatic and breast cancer and may affect the health of their family(1, 2). Most of these men are currently unaware that they have a cancer-predisposing *BRCA* variant, but as genetic testing becomes more common, more men will need medical advice about what having such a variant means for them and their families.

Men are just as likely as women to have a cancer-predisposing *BRCA* variant, but many people perceive these variants as only being relevant to women. Paradoxically, this could lead to women at very high risk of breast and ovarian cancer missing out on screening and risk-lowering treatment despite a concerning paternal family history. Clinicians might also be less attuned to paternal family history of cancer in assessing women’s breast cancer risk(3).

This practice pointer covers what cancer-predisposing *BRCA* variants are; who might be tested; and what health issues men and their clinicians need to know about. We refer to men but this article may also apply to some transgender and non-binary people: their risk profiles and recommended care should be appropriately individualised.

## What are cancer-predisposing *BRCA* variants?

*BRCA1* and *BRCA2* are tumour suppressor genes that code for DNA repair proteins. Certain variants in these genes predispose to cancer (primarily breast, ovarian, prostate and pancreatic, and for *BRCA2* possibly melanoma(4)). The predisposition to cancer is inherited in an autosomal dominant way i.e. each and every time a person with a cancer-predisposing *BRCA* variant has a child, they have a 1 in 2 or 50:50 chance of passing their *BRCA* variant onto the child. This is the case regardless of whether parent or child is male or female.

**Figure 1:**
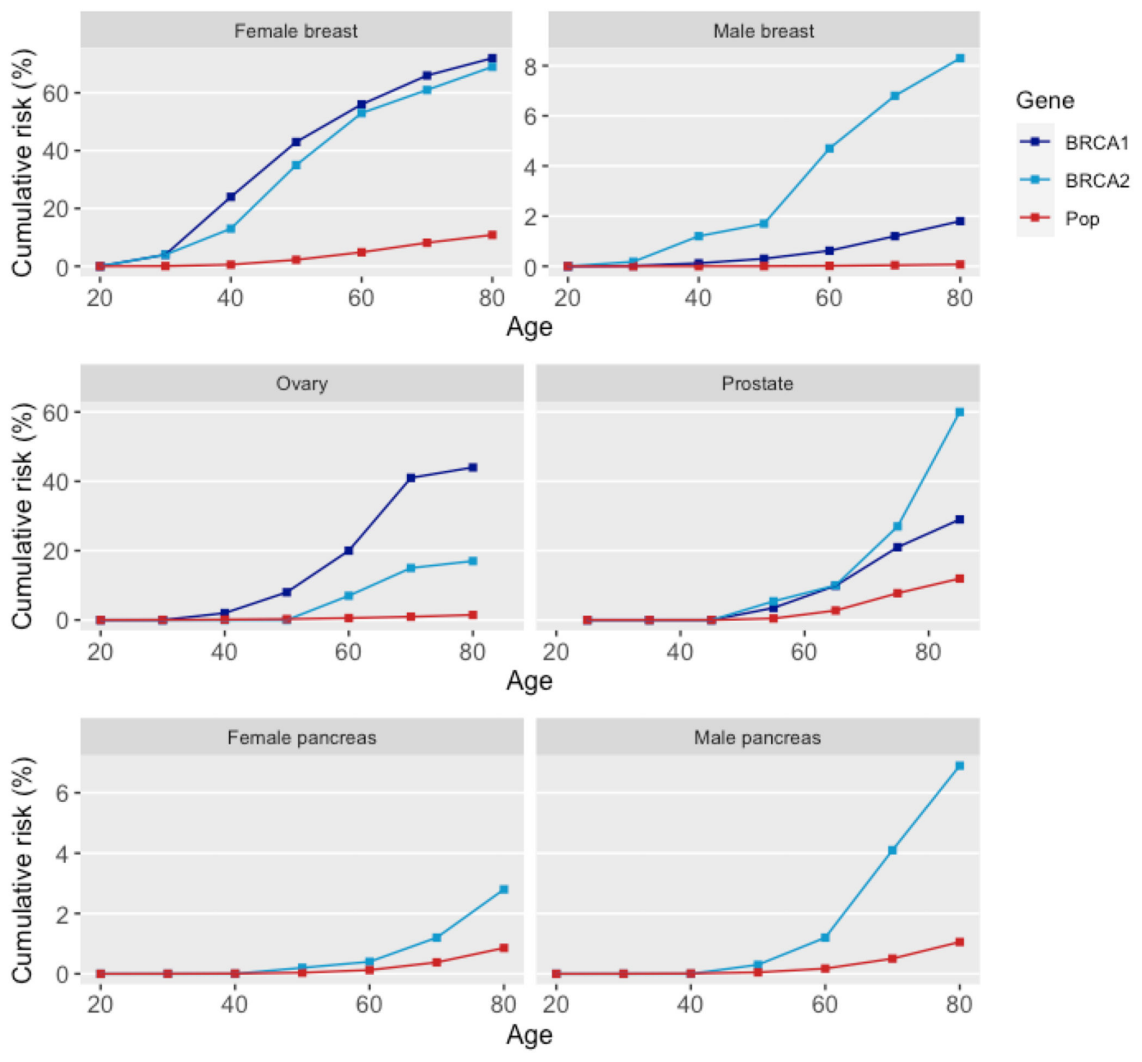
Cancer risks associated with cancer-predisposing *BRCA* variants Please note cumulative cancer risks are shown on different scales *Cancer-predisposing *BRCA1* variants also increase pancreatic cancer risk (relative risk 4.11)(8) but data are emerging and cumulative risk figures are not readily available. Female breast and ovary data from Kuchenbaecker *et al*. 2017(9); prostate from Nyberg *et al*. 2020(10); male breast from Tai *et al*. 2007(11); and pancreatic cancer from van Asperen *et al*. 2005(12). Population data are for England and Wales 2016 (from Office for National Statistics).

Many men and some women with cancer-predisposing *BRCA* variants will never develop an associated cancer, as shown in Figure 1. The cancer risks associated with cancer-predisposing *BRCA* variants are modified by lifestyle factors and other inherited genetic variants. Polygenic risk scores (calculated by looking at multiple common genetic variants across the genome, each with a tiny individual effect) show potential to refine cancer risk predictions for people with cancer-predisposing *BRCA* variants, but cannot remove uncertainty as to whether a given patient will or will not develop cancer(5).

Thousands of different variants within the *BRCA1* and *BRCA2* genes have been described, and classifying which are benign and which increase cancer risk can be challenging(6). As more data are gathered, classifications may shift. For example a Canadian laboratory examined the *BRCA* variants they identified over a five year period and found that 12% were reclassified (75% of these were downgraded – i.e. they are now thought to be less likely to predispose to cancer than they were when the *BRCA* test was originally done)(7). Diagnostic tests involve sequencing of *BRCA1* and *BRCA2* (and perhaps other genes, e.g. *PALB2*), aiming to detect any variant present that might increase cancer risk.

## How would a man find out that he has a cancer-predisposing *BRCA* variant?

In the UK, diagnostic *BRCA* testing is currently offered to people with a 10% or greater chance of having a cancer-predisposing *BRCA* variant(15). Most diagnostic *BRCA* tests are done for women, but some men will find out their *BRCA* status via this route, as men with breast cancer at any age are eligible to be tested(16), and some men with prostate or pancreatic cancer may have testing to determine eligibility for PARP inhibitors (often in the context of clinical trials).

More commonly, men are offered targeted *BRCA* testing after a cancer-predisposing *BRCA* variant is identified in their family. Usually this will be predictive testing (i.e. the man has no personal medical history suggestive of a cancer-predisposing *BRCA* variant), though occasionally it may be explanatory (e.g. in men known to have prostate cancer). The laboratory would need details of the specific cancer-predisposing *BRCA* variant in the man’s family in order to test for it (e.g. a genetic report from an affected family member) and such targeted testing would not detect any other cancer-predisposing variants.

Around 1.5% of patients with prostate cancer have a cancer-predisposing *BRCA* variant(17), but a personal medical history of prostate cancer is not an indication for diagnostic *BRCA* testing in the UK. However, for patients with younger-onset, aggressive disease, a thorough family history can explore the possibility of a familial cancer-predisposing *BRCA* variant. A family history that includes, for example, bilateral breast cancer, male breast cancer, or multiple people affected by breast or ovarian cancer (especially at younger ages), probably warrants discussion with your local genetics service.

Some men might access *BRCA* testing outside standard clinical pathways, such as through direct-to-consumer genetic testing or research studies(18). These tests are of variable quality and scope, and further scrutiny may be needed to confirm that the variant is really present or really represents a risk(19).

## Case studies

### Cancer-predisposing *BRCA* variants increase the risk of aggressive prostate cancer

*Rahul is a 40-year-old man who had a test via a genetics clinic for the cancer-predisposing BRCA2 variant identified in his aunt. Rahul was also found to have the cancer-predisposing BRCA2 variant and is concerned about developing prostate cancer. He books an appointment with you to discuss PSA screening.*

Men with cancer-predisposing *BRCA2* variants like Rahul have an increased risk of prostate cancer: a recent meta-analysis indicated an odds ratio of 2.64(17), and a large prospective cohort study in the UK and Ireland found a 27% absolute risk of developing prostate cancer by age 75, rising to 60% by age 85(10) (for comparison, population risk by age 85 is 16% in England and Wales based on Office for National Statistics 2016 data). When prostate cancer does occur in a man with a cancer-predisposing *BRCA2* variant, it tends to be more aggressive(20). The evidence has been inconsistent regarding the impact of cancer-predisposing *BRCA1* variants on prostate cancer but a subtler effect is probable, with an odds ratio of 1.35(17), and the relative risk increase is higher at younger ages(21, 22).

The IMPACT study is an ongoing international prospective cohort study of more than 3,000 men to examine the use of PSA screening in men with cancer-predisposing *BRCA* variants. Based on interim results, the researchers recommend that men with cancer-predisposing *BRCA2* variants are offered systematic PSA screening(23). This is because after three years of PSA screening, men with cancer-predisposing *BRCA2* variants proved to have a higher incidence of prostate cancer, were younger at diagnosis, and were more likely to have clinically significant tumours. The positive predictive value of a PSA >3.0 ng/ml was higher in men with cancer-predisposing *BRCA2* variants than in controls (31% vs 18%).

Discuss with Rahul the pros and cons of PSA screening and be clear about the current limits of medical knowledge. It would be appropriate to include in this discussion that if prostate cancer does develop in a man with a cancer-predisposing *BRCA2* variant, it is more likely to be clinically significant. Interim analysis from the IMPACT study shows that after four screening rounds (annual PSA) for men aged 55-69 with cancer-predisposing *BRCA2* variants, you would expect to detect one clinically significant prostate cancer for every 13 men screened(23). The European Association of Urology recommends offering PSA based prostate cancer screening to men with cancer-predisposing *BRCA2* variants who have been counselled on the potential risks and benefits of screening from the age of 40 although they do not specify a screening interval(24). It is not yet known whether PSA screening will reduce mortality from prostate cancer in men with cancer-predisposing *BRCA2* variants.

Research is ongoing as to the role of PSA screening for men with cancer-predisposing *BRCA1* variants: an interim analysis found no differences in age or tumour characteristics between men with cancer-predisposing *BRCA1* variants and controls(23).

### Breast awareness is important for men with cancer-predisposing *BRCA* variants

*Jakob is a 50-year-old man who books an appointment to discuss a painless ‘cyst’ near his left nipple that he noticed several months ago. On examination, you notice that he has an inverted nipple and ipsilateral axillary lymphadenopathy. You ask about his family history and he tells you that his father died of prostate cancer in his sixties*.

Men with cancer-predisposing *BRCA* variants have an increased lifetime risk of developing breast cancer: 8.3% for *BRCA2* and 1.8% for *BRCA1*, compared to 0.1% in the general population(11). As in women, breast cancer in men most commonly presents as a painless mass. Although nipple involvement tends to be seen earlier due to the smaller amount of breast tissue(25), male breast cancer is often diagnosed at an advanced stage(25). Men with cancer-predisposing *BRCA* variants are at higher risk of developing breast cancer, but any man with symptoms of breast cancer warrants urgent referral to a breast clinic in the same way women presenting with concerning symptoms would be referred, regardless of *BRCA* status.

Evidence regarding breast cancer characteristics in men with cancer-predisposing *BRCA* variants is very limited. Two studies analysing tumour grading, staging and receptor status in men with breast cancer suggest that cancer-predisposing *BRCA2* variants are associated with more aggressive cancers(26, 27).

Evidence is also lacking on breast cancer screening in men with cancer-predisposing *BRCA* variants and practice varies(28). In the UK, men with cancer-predisposing *BRCA* variants are advised to be breast aware, i.e. to know how their breasts usually look and feel, and seek medical advice if they notice changes or have any concerns. Men might be more likely to delay seeking care for a new breast lump, perhaps waiting until the lump becomes painful or changes the overlying skin. A study in Hong Kong of men with breast cancer found that median duration from symptoms to first medical consultation was 12.4 months, and 84% were not aware (before their diagnosis) that breast cancer could occur in men(29). Raising this issue may be challenging, particularly since resources promoting breast awareness are mainly aimed at women(30). Our hospital Patient and Public Involvement group highlighted some of the issues men might face during and after receiving a breast cancer diagnosis, for example being the only man in the waiting room for appointments.

### Patients may be unaware that cancer-predisposing *BRCA* variants can be passed on by men

*Harry is a 55-year-old man who you see regularly regarding his poorly controlled diabetes. At the end of an appointment discussing his blood sugars, he mentions that his sister in Australia has recently told him that she “has BRCA” and that he should get tested. You ask how he feels about this and he says he can’t see the point because “isn’t BRCA a female thing?” Harry has two daughters in their thirties*.

Because cancer-predisposing *BRCA1* and *BRCA2* variants are notorious for increasing lifetime risk of breast and ovarian cancer in women, there is a common misperception that cancer-predisposing *BRCA* variants themselves only occur in women(31). Cancer-predisposing *BRCA* variants are just as common in men as they are in women, but are less likely to be detected because they are less likely to cause a cancer that prompts genetic testing.

Explain that men and women inherit these genetic variants in the same way: that *any* child of a parent with a cancer-predisposing *BRCA* variant has a 50:50 chance of inheriting that variant. This would include Harry’s children if Harry also has the *BRCA* variant identified in his sister.

For some men, concern for the health of existing or potential future daughters or granddaughters is a key motivation for seeking *BRCA* testing(31, 32). In contrast, sometimes men are reluctant to have *BRCA* testing because they are concerned about potentially having passed a cancer-predisposing *BRCA* variant onto their children. They might prefer not to know than to have this possibility confirmed. It may help to remind them that the cancer-predisposing *BRCA* variant has likely been in their family for generations and whether they inherited or passed it on is outside their control.

**Figure 2:**
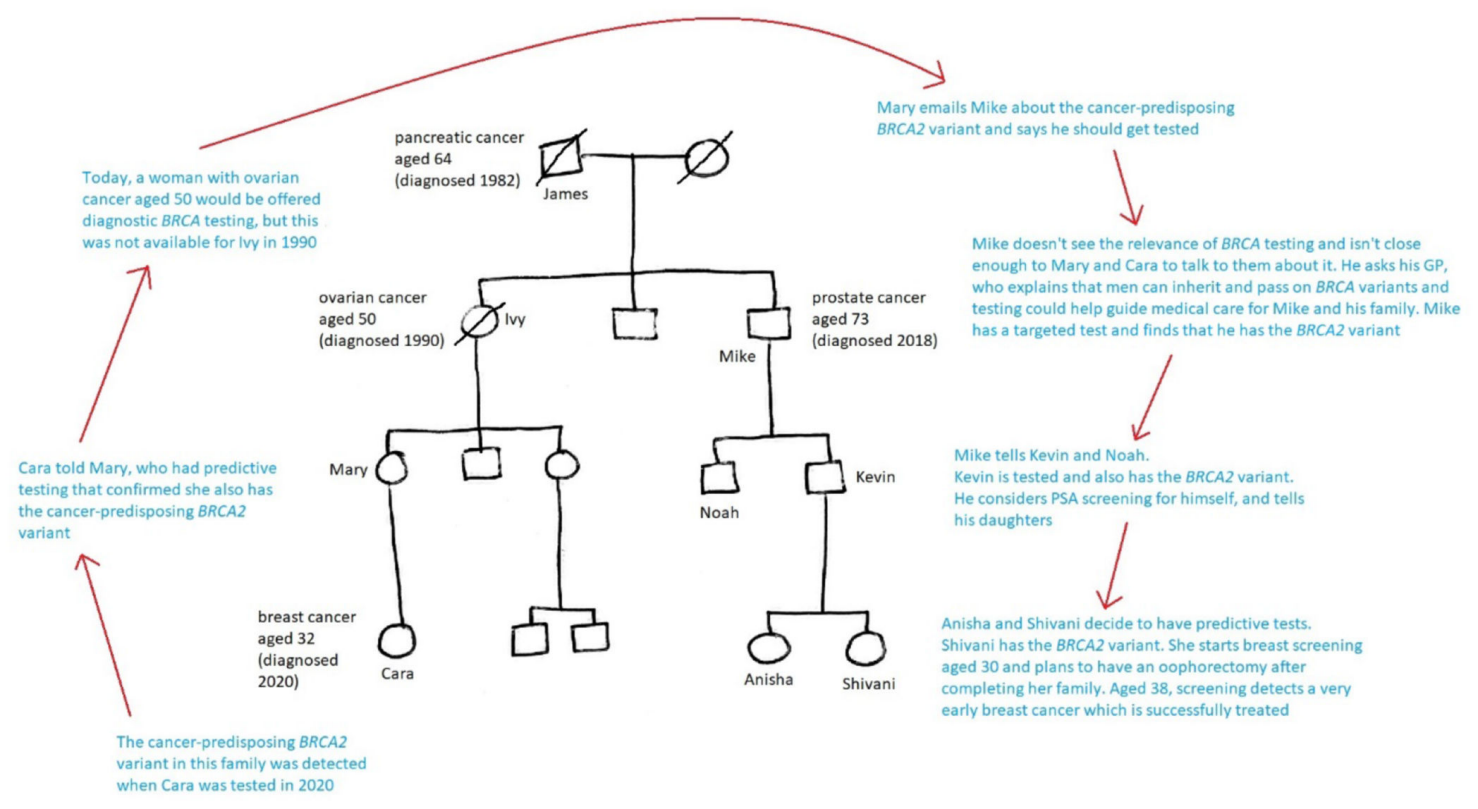
A family learning about *BRCA*

However, being tested for it might guide medical care for them and their children, for example by informing choices about cancer screening (Figure 2).

Occasionally, men decide that they do not wish to be tested for the cancer-predisposing *BRCA* variant found in their family. It would still be appropriate and important to refer their adult, first degree relatives (e.g. siblings or children) to clinical genetics specialists if they wish, even if testing them might (indirectly) reveal that the man has the cancer-predisposing *BRCA* variant. Clinical genetic services are skilled at counselling patients through difficult psychosocial and ethical issues such as this scenario.

### Men with cancer-predisposing *BRCA* variants have an increased risk of developing pancreatic cancer

*Simon is a 50 year old man who had a test two years ago via a genetics clinic for the cancer-predisposing BRCA2 variant identified in his sister. Simon was also found to have the BRCA2 variant and discussed his increased risk of prostate and pancreatic cancer with a genetic counsellor at the time. Last week, Simon’s friend was diagnosed with pancreatic cancer. Simon is now more concerned about his risk of pancreatic cancer and books an appointment to discuss it with you*.

People with cancer-predisposing *BRCA* variants have an increased risk of developing pancreatic cancer: a retrospective cohort analysis of a high-risk breast cancer family registry from the US, Canada and Australia estimated that pancreatic cancer risk is five to six times higher than population risk for people with cancer-predisposing *BRCA2* variants and around four times higher for people with cancer-predisposing *BRCA1* variants(8). Unfortunately pancreatic cancer is challenging to screen for and often advanced by the time symptoms develop. 2018 NICE guidance on pancreatic cancer recommended that people with cancer-predisposing *BRCA* variants and one or more first degree relatives with pancreatic cancer have surveillance (MRI/MR cholangiopancreatography or endoscopic ultrasound)(33), but this was challenged by the UK Cancer Genetics Group as being premature(34). Instead, the group recommended that pancreatic cancer screening should only be offered within the context of research studies such as EUROPAC(35). In practice, pancreatic cancer screening is generally considered on a case-by-case basis for people with cancer-predisposing *BRCA* variants if they also have a family history of pancreatic cancer. Screening as part of a research study might be a possibility for Simon – your local genetics service may be able to signpost towards this.

This consultation could be a good opportunity to discuss lifestyle factors – people cannot change whether they have a cancer-predisposing *BRCA* variant, but addressing modifiable factors like smoking and alcohol intake will reduce their risk of developing cancer.

Around 6-7% of people with metastatic pancreatic cancer have a germline cancer-predisposing *BRCA* variant(36). Increasingly, people with pancreatic cancer are offered diagnostic *BRCA* testing in order to inform treatment plans. Cancer cells with cancer-predisposing *BRCA* variants already have impaired DNA repair and are heavily reliant on DNA repair pathways involving PARP, so are particularly vulnerable to PARP inhibitors. PARP inhibitors may be a treatment option for people with a germline cancer-predisposing *BRCA* variant who have breast, ovarian, pancreatic or prostate cancer (often in the context of clinical trials)(37).

**What you need to know**

- Men and women are equally likely to inherit or pass on a cancer-predisposing *BRCA* variant – family history of cancers needs to encompass both sides of the family.
- Men with cancer-predisposing *BRCA* variants have an increased risk of developing breast cancer and are advised to be breast aware.
- Men with cancer-predisposing *BRCA2* variants have an increased risk of developing aggressive prostate cancer. Evidence is less clear regarding men with cancer-predisposing *BRCA1* variants but they probably also have an increased risk. We don’t yet know whether prostate specific antigen screening reduces mortality in men with cancer-predisposing *BRCA* variants.
- The European Association of Urology recommends that PSA screening is offered to men with cancer-predisposing *BRCA2* variants from 40 years of age after discussion of the risks and benefits.

**How patients were involved in the creation of this article**

We spoke with men from the Patient and Public Involvement group at University Hospitals Southampton NHS Foundation Trust to discuss what prior knowledge men might have about cancer-predisposing *BRCA* variants, what information they might want to know after finding that they had a cancer-predisposing *BRCA* variant, and what terminology they might prefer when talking about genetic variants. These discussions particularly influenced the case study section, the discussion about expectations in ‘How would a man find out that he has a cancer-predisposing *BRCA* variant?’, and Box 1: *Describing genetic variants*.

**Education into practice**

- Do you ask about paternal as well as maternal family history when assessing breast cancer risk?
- What questions might you ask if a man tells you he doesn’t want testing for the cancer-predisposing *BRCA* variant found in his family?
- When did you last ask a man about his family history of breast and ovarian cancer?

**How this article was made**

This article was commissioned by the education team. We developed fictitious cases to illustrate common issues that may arise for men with cancer-predisposing *BRCA* variants, based on scenarios encountered by our regional genetics department.

We used PubMed, and author research paper archives, to search for information about cancer risks and medical care for men with cancer-predisposing *BRCA* variants. Where available, we aimed to quote cancer risks established by large prospective cohort studies. Medical care of men with cancer-predisposing *BRCA* variants is under-researched and often guidelines are lacking – in these cases, we have drawn on our context within a UK regional genetics department to aim to reflect UK standard practice.

**Ethical issues**

Identification of a cancer-predisposing *BRCA* variant may raise various ethical issues, for example:

- ***Sharing of genetic information within families***
  Family members may benefit from testing for the cancer-predisposing *BRCA* variant. What if the person in whom it was identified finds it difficult to tell their family about the variant, or chooses not to? Health professionals may sometimes need to balance the competing tensions of patient confidentiality with the interests others have in knowing about their risks.
- ***Decisions around termination of pregnancy due to adult-onset conditions***
  People with cancer-predisposing *BRCA* variants have a substantially increased risk of developing certain cancers, but these are adult-onset and some people with such variants will never develop cancer.
- ***Shifting understanding of what particular genetic variants mean***
  Classifying *BRCA* variants as benign or cancer-predisposing is technically challenging. Over time, new evidence may shift our understanding of what a particular variant means (i.e. it may become clear that a variant thought to predispose to cancer is actually benign, or vice versa). How should clinicians respond when this happens?

## Box 1 Describing genetic variants

Various terms are used to describe genetic variants that increase risk of disease. As with many medical terms, different people might understand different things by the same term.

The term ‘mutation’ is often used in patient leaflets, patient support forums, and in UK school curricula. However, some find the term offensive(13) and it is technically incorrect since it suggests the mutation has happened in that person. ‘Faulty gene’ is also often used in patient resources, and a 2007 Australian survey indicated that this term was most preferred by the general community and by patients recruited via cancer genetics clinics(14).

‘Pathogenic or likely pathogenic variant’ is commonly used in laboratory genetic reports. People are increasingly familiar with the term ‘variant’ in the context of covid-19, but ‘pathogenic’ may be less well understood by people without a healthcare background, so ‘disease-causing’ is sometimes used instead. However, this in turn may give the false impression that everyone with a ‘disease-causing *BRCA* variant’ will inevitably develop an associated cancer.

Other terms in common use include: ‘disease-risk variant’; ‘genetic risk factor’; ‘disease-predisposing variant’; ‘loss-of-function variant’; ‘gene alteration’; ‘gene change’, and for people with cancer-predisposing *BRCA* variants, ‘*BRCA* carriers’. In discussion with our hospital Patient and Public Involvement group it was evident that different people found different terms most helpful. We use the term ‘cancer-predisposing variant’ here as it is informative and technically accurate, but whatever term you use to describe cancer-predisposing *BRCA* variants, key messages to convey are: (1) they are very likely to be inherited rather than to have happened for the first time in the tested person; and (2) they are *risk factors* for particular cancers rather than certainties that they will develop.
